# Clinical features and prognosis of parotid metastasis of breast cancer: retrospective analysis of 57 cases

**DOI:** 10.3389/fonc.2024.1442713

**Published:** 2024-09-02

**Authors:** Fengli Guo, Honghai Fu, Yuhua Wang, Yitong Hua, Xiaohong Wang, Yingzhe Zhang, Jinbo Jian, Zhongming Jia, Guoqiang Zhang

**Affiliations:** ^1^ Department of Thyroid and Breast Surgery, Binzhou Medical University Hospital, Binzhou, China; ^2^ Department of Oral and Maxillofacial Surgery, Binzhou Medical University Hospital, Binzhou, China; ^3^ Department of Oncology, Binzhou Medical University Hospital, Binzhou, China

**Keywords:** breast cancer, parotid, concomitant metastases, prognosis, overall survival

## Abstract

**Purpose:**

Parotid gland metastases originating from breast origin are extremely rare, with their clinical presentation, therapeutic approaches, and prognostic indicators remaining to be elucidate.

**Methods:**

A comprehensive retrospective review was conducted, analyzing the clinical characteristics and prognostic factors of 57 patients diagnosed with parotid metastasis of breast cancer in the existing literature. Notably, our study included two unique cases of patients who developed contralateral and ipsilateral parotid metastases, occurring 5 years and 32 years respectively after primary surgery. This analysis aimed to provide a deeper understanding of the disease presentation and identify potential prognostic indicators.

**Results:**

The primary clinical manifestation presented in breast cancer patients with parotid metastases was painless masses in the parotid glands, synchronously or metachronously occurred with primary breast tumors. The predominant pathological subtype among these patients was invasive ductal carcinoma. Out of the 57 patients studied, 24 (42.1%) exhibited metastases solely in the ipsilateral parotid gland, while 18 cases (31.6%) involved either the contralateral or bilateral parotid gland. Patients may solely exhibit metastasis in the parotid gland, or they may present with concurrent multiple metastases in other organs. Patients who suffered from parotid metastases, either merely or accompanied with bone-only metastasis, exhibited significantly longer overall survival (OS) rates compared to those who had concomitant metastases in other organs (1.23 ± 0.26 years vs 4.46 ± 0.77 years, P=0.046). While no statistically significant differences in OS were observed among patients presenting with metastases in the ipsilateral, contralateral, or bilateral parotid glands, a notable variance could be discerned from the Kaplan-Meier curve analysis. Additionally, no significant difference in survival was exhibited between patients with different interval of progression from primary breast sites to initial diagnosis of parotid metastases (uDF), nor for patients who were treated with surgery or palliative therapy.

**Conclusion:**

Parotid metastasis, a rare and distinctive form of breast cancer metastasis, demands particular scrutiny in patients exhibiting metastasis to multiple organs or contralateral or bilateral parotid glands.

## 1 Introduction

The parotid gland is an unusual site for metastatic disease and when metastasis occurs, it commonly originates from head and neck primaries. Malignant melanoma and squamous cell cancer are the two most common tumors which metastasize to the parotid gland (1). Parotid gland metastases are rarely from any source other than the head and neck (2), due to differences in the number of lymph nodes, anatomical relationships, and their drainage (3). Metastasis of breast cancer to the parotid gland is an exceedingly rare occurrence, which can manifest synchronously or metachronously, even years after the initial diagnosis, irrespective of primary disease stage and appropriate primary treatment. In 1975, Katz described a metastatic adenocarcinoma from the left breast to the right parotid gland (4). Since then, only occasional studies have reported this specific metastasis. Although the morbidity is low, the mortality rate is quite high. Two previous studies have retrospectively described parotid metastasis of breast cancer and reviewed the literature, however, the literature included is insufficient, and the incidence, clinical presentation, management and prognosis of the disease are not adequately described (5, 6).

In this article, we presented two patients with a history of an invasive ductal carcinoma who developed contralateral and ipsilateral parotid metastases 5 and 32 years after surgery, respectively, as a first step. Subsequently, a retrospective review of 57 patients diagnosed with parotid metastasis of breast cancer in the literature was conducted to describe the presentation, management strategies and investigate the prognostic factors. To our knowledge, this is the first comprehensive analysis regarding to the clinical features and prognostic factors of parotid gland metastasis from breast cancer.

## 2 Materials and methods

Firstly, we initially presented 2 cases of parotid gland metastasis of breast cancer patients in our hospital. Secondly, a comprehensive literature search of the PubMed and Medline databases with key words including “breast cancer”,”breast carcinoma” AND “parotid” to identify studies of parotid metastasis from breast cancer published from 1975 to 2023 was performed, including articles provided in literature. A total of 42 articles and 57 patients were included. Detailed information about these patients were retrieved from these articles. Thirdly, the descriptive analyses of clinical characteristics and statistical analyses of prognostic factors associated with parotid metastasis of breast cancer were conducted.

### 2.1 Statistical analysis

Data were analyzed using the SPSS software (for Windows, version 22.0; IBM Corp., Armonk, NY, USA). Kaplan–Meier and log-rank tests were used to estimate the overall survival (OS). Two-tailed *P* values <0.05 denoted statistically significant differences.

## 3 Results

### 3.1 Case 1

A 58-year-old female patient presented with the concern of a painless lump in the right parotid region, accompanied by discomfort on the corresponding side of her face in April 2022. Upon clinical examination, a firm yet painless mass was detected in the right parotid region, exhibiting no signs of inflammation or facial nerve paralysis.

**Figure 1:**
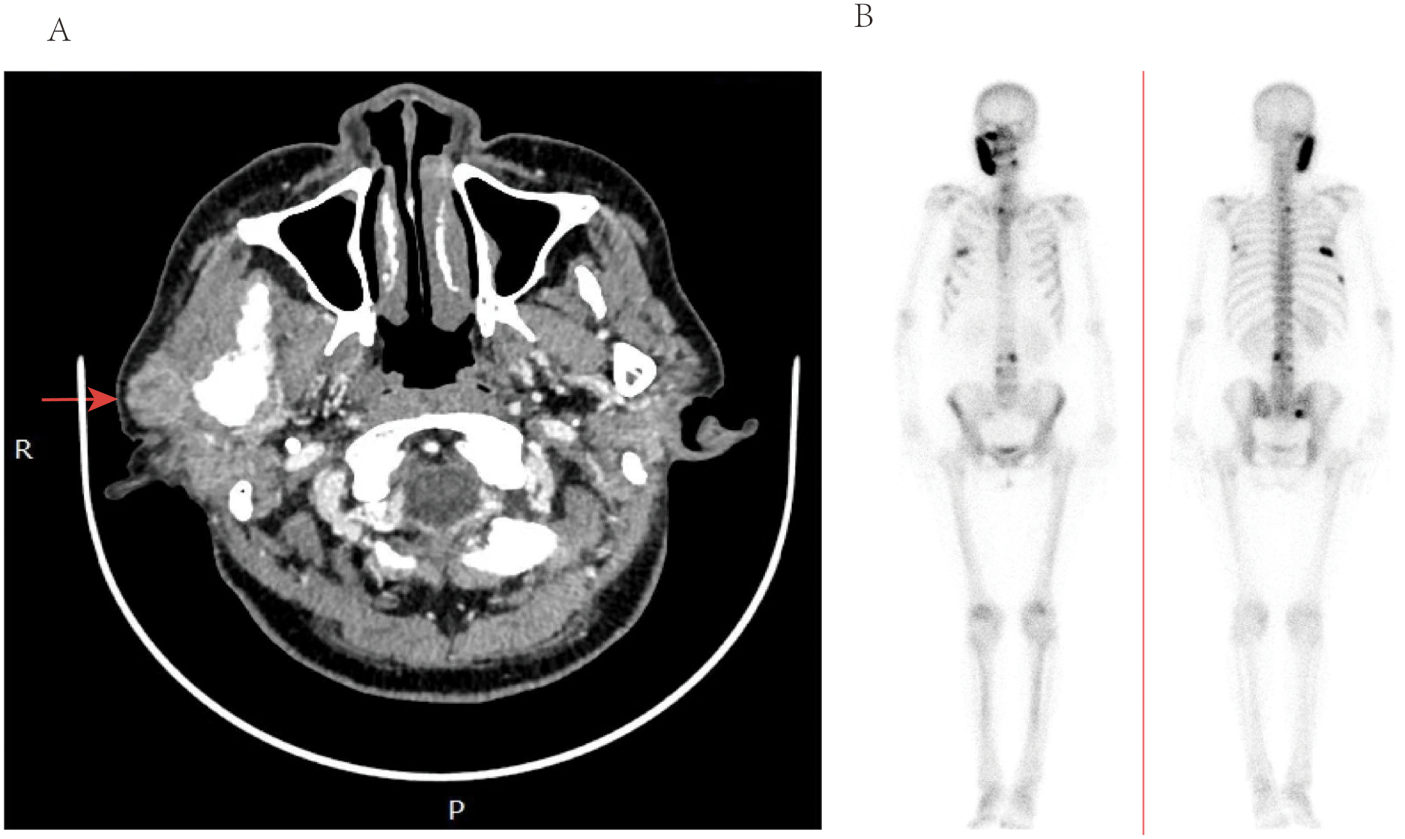
**(A)** Computed tomography of the parotid with contrast—axial view, demonstrating heterogenous enhancement and enlargement of the right parotid glands. **(B)** ECT imaging, demonstrating marked multiple lesions with increased uptake of imaging agents in ribs and vertebrae.

The computed tomography (CT) scan with contrast revealed a distinct, heterogeneous, round enhancement in the superficial lobe of the parotid gland, measuring approximately 1.5cm x 1.8cm in size. Notably, the bone of the right mandibular ramus appeared thickened, whereas the adjacent soft tissue appeared unremarkable (see **Figure 1A**). Furthermore, the neck, submandibular, and submental regions exhibited bilateral cervical lymph nodes, all measuring below the centimeter range. Upon further questioning, the patient revealed a past diagnosis of infiltrating ductal carcinoma (IDC) in the left breast, occurring approximately five years ago. The clinical staging at that time was designated as T2N1M0. She underwent a comprehensive treatment strategy including mastectomy and axillary lymph node dissection, adjuvant chemotherapy and loco-regional radiotherapy followed by a five-year course of hormone therapy to ensure optimal recovery and reduce the risk of recurrence.

The patient underwent resection of the mass in the superficial lobe of the right parotid gland, and intraoperative pathological examination subsequently revealed the presence of metastatic breast cancer cells. Additionally, a biopsy conducted on the left mandible did not demonstrate any evidence of malignant tumor metastasis, indicating that the cancer had not spread to this region. No local biopsy or radiotherapy was performed on the subcm nodes in bilateral cervical with a consideration of non-metastases.

**Figure 2:**
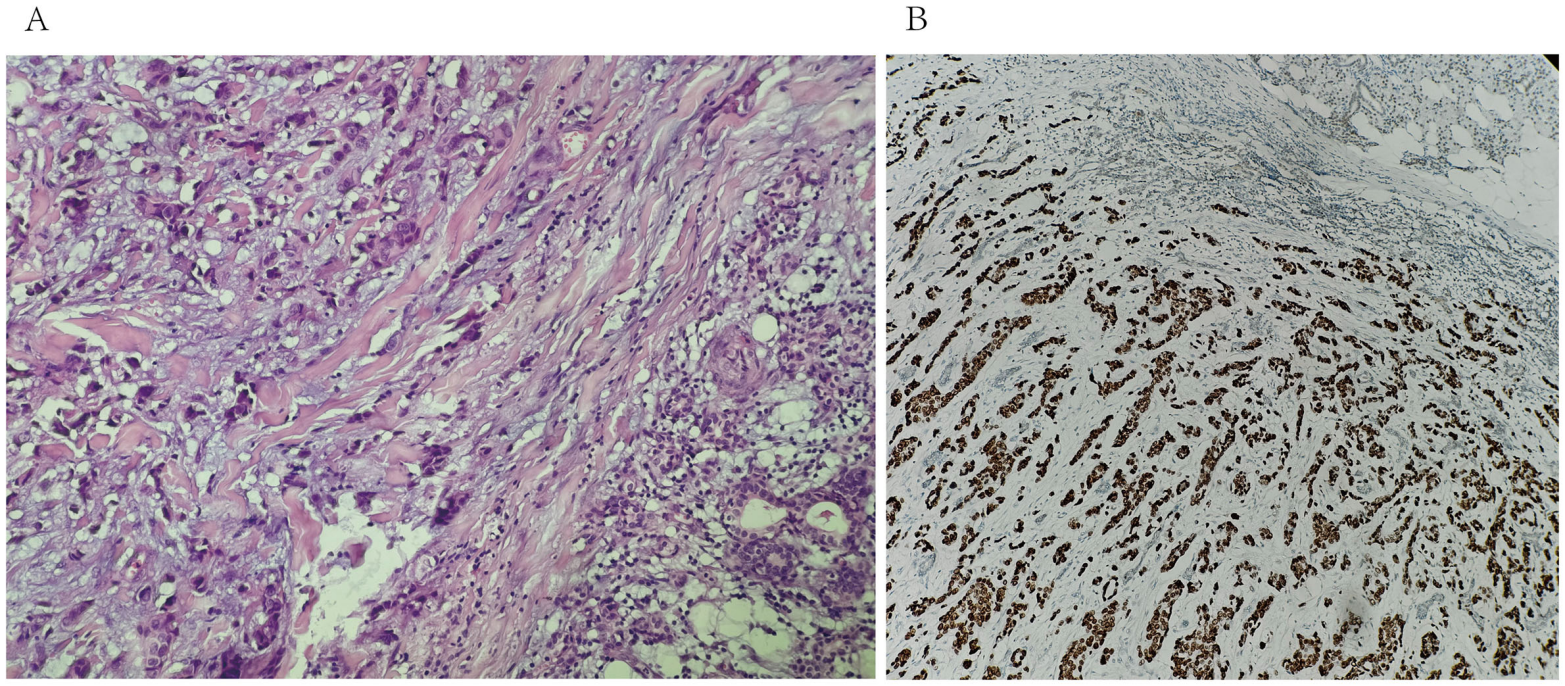
**(A)** biopsy of the right parotid mass with Hematoxylin & Eosin staining (20X) shows tumoral cells in the salivary gland. **(B)** immunohistochemistry for GATA-3 shows positive staining of tumor cells (20X).

Subsequently, a superficial parotidectomy was executed. Immunohistochemical analysis revealed a positive expression of estrogen receptor (ER), progesterone receptor (PR), GATA-3, and human epidermal growth factor receptor 2 (HER-2) with a high score of 3+. **Figure 2** depicted the HE staining and GATA-3 staining images of this particular patient. Additionally, the monoclonal antibody Ki-67 index indicated a proliferative rate of 30%. Furthermore, emission computed tomography (ECT) scans confirmed the presence of multiple metastases in the ribs and vertebrae, as depicted in **Figure 1B**. An extensive treatment regime was carefully devised to ensure optimal care for the patient. Subsequently, the patient underwent several rounds of adjuvant chemotherapy, which was complemented by targeted therapy employing trastuzumab and pertuzumab. Radiotherapy was administered as the next phase of treatment, followed by hormone therapy to further consolidate the therapeutic gains. Over the past two years, the patient experienced a notable remission from the disease, but unfortunately, there has been a recent localized recurrence in the right sternocleidomastoid muscle.

### 3.2 Case 2

**Figure 3:**
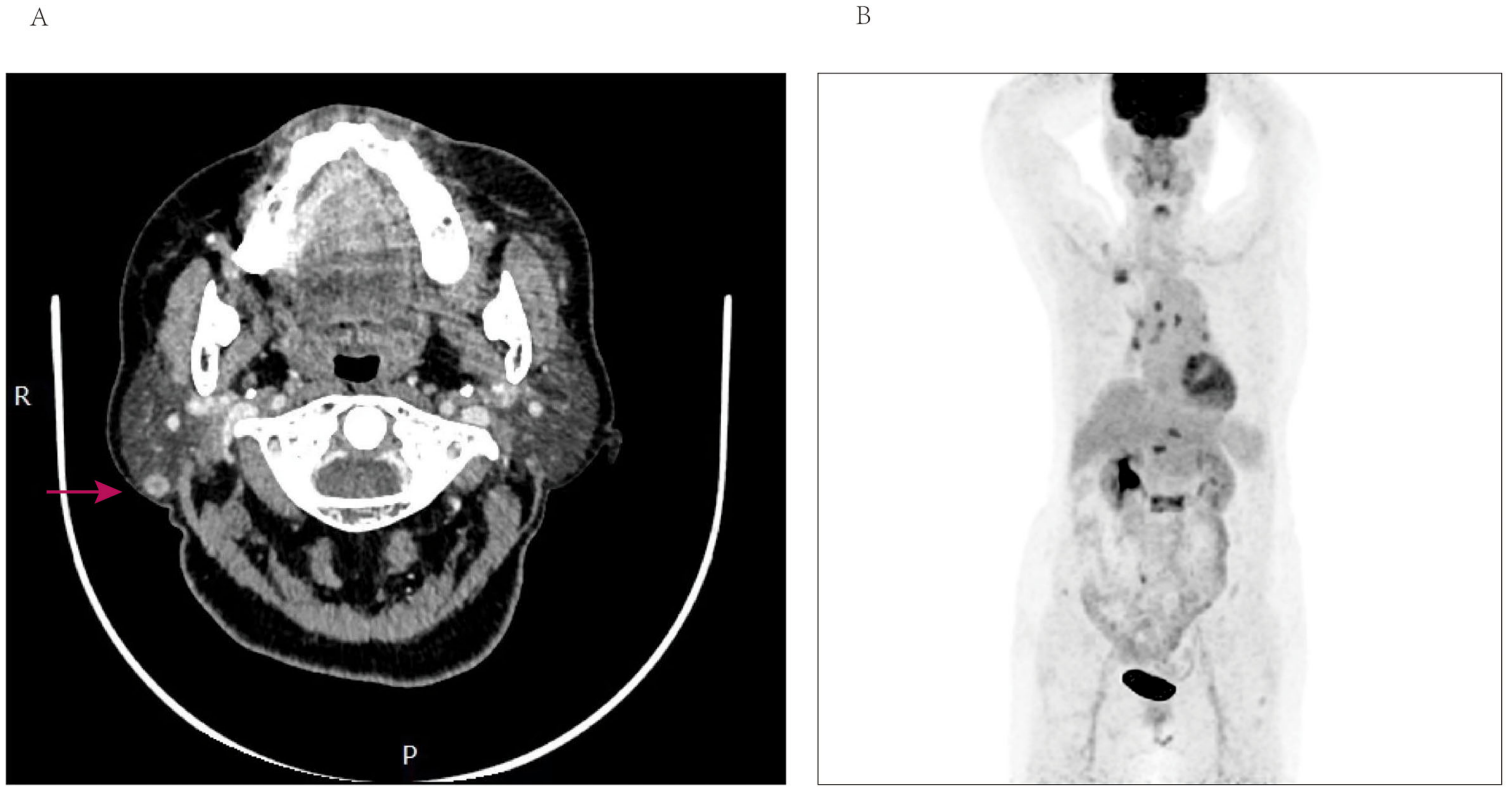
**(A)** Computed tomography of the parotid with contrast—axial view, demonstrating well-demarcated enhancement nodular of the right parotid gland. **(B)** PET-CT scan reveals accumulation of SUV more than 8 in multiple vertebrae, ribs, the right ilium, and lymph nodes.

In March 2023, a 71-year-old female patient presented with a one-month history of a painless lump behind her right ear. A parotid CT scan with contrast enhancement confirmed the existence of a 0.7-centimeter, clearly defined nodular mass (**Figure 3A**). Notably, this patient had undergone surgery for right breast cancer approximately 32 years prior to her current presentation. Additionally, a chest CT scan revealed abnormalities in multiple vertebrae and ribs, suggestive of bone involvement.

**Figure 4:**
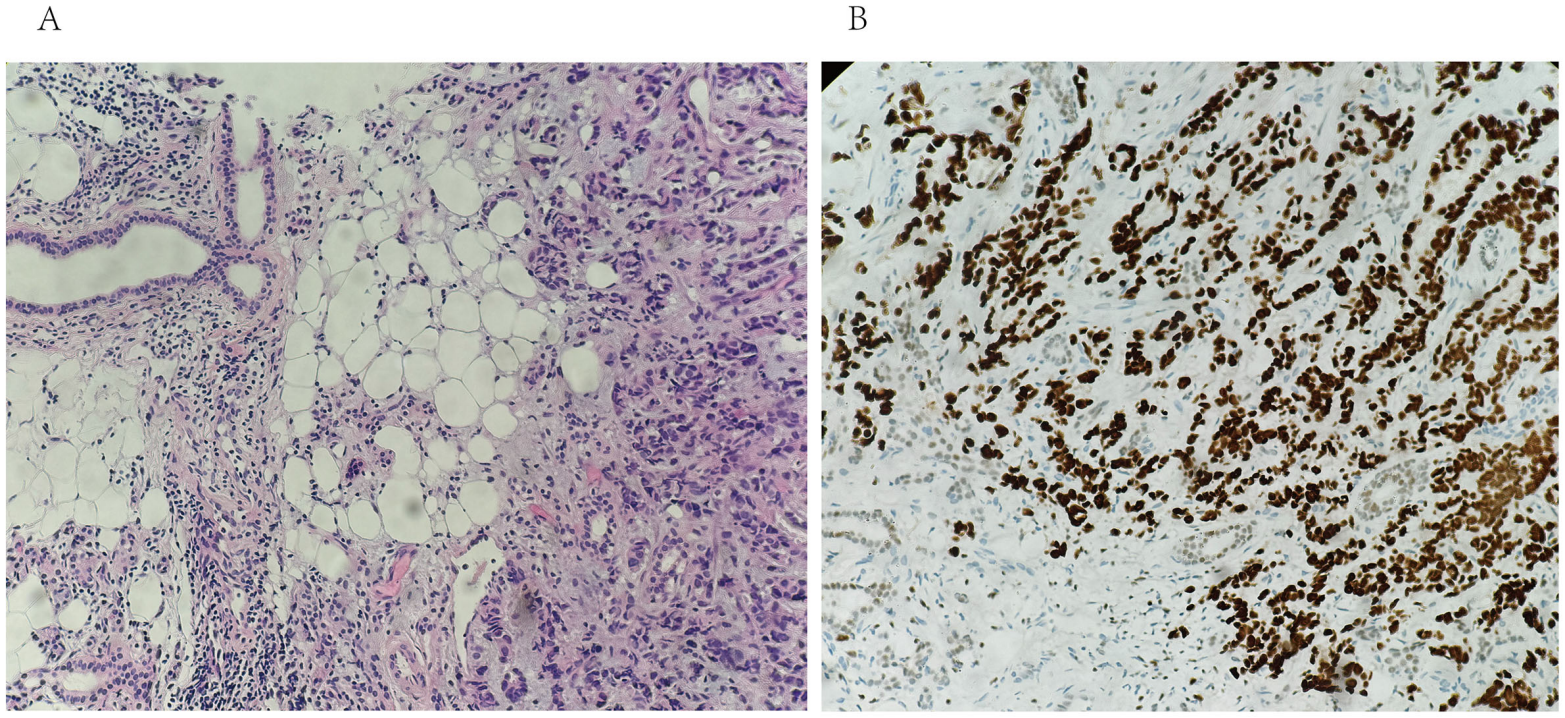
Core needle biopsy of the right parotid mass with Hematoxylin & Eosin staining (20X) shows tumoral cells in the salivary gland. **(B)** immunohistochemistry for GATA-3 shows positive staining of tumor cells (20X).

Taking into account the patient’s medical history and the observed findings, the possibility of metastases was seriously considered. Consequently, a core needle biopsy was promptly conducted in the parotid region. Pathological analysis indicated the presence of metastatic breast cancer, while immunohistochemical studies revealed positive expression of estrogen receptor (ER), GATA-3 and negative expression of progesterone receptor (PR). Additionally, the Ki-67 index was determined to be 30%, indicating a moderate proliferative activity. HER-2 staining was recorded as 2+, but FISH testing yielded negative results. **Figure 4** depicted the HE staining and GATA-3 staining images of this patient. The results of positron-emission tomography CT (PET-CT) revealed multiple osteoblastic bone changes in the skull base, multiple vertebrae, the right first rib, the left sixth rib, the right ilium. The patient exhibited a significant accumulation of standardized uptake value (SUV max) in the L2 vertebrae, reaching 9.3, and a similar uptake of 8.8 in the right parotid gland. Furthermore, the PET/CT scan revealed multiple metastases in the lymph nodes, including those located in the right supraclavicular region, mediastinal area, and bilateral hilar regions (**Figure 3B**). Subsequently, the patient underwent hormone therapy with a combination of palbociclib and fulvestrant. Notably, the patient has maintained clinical stability up to the present time, and there has been no detection or confirmation of recurrence at any other sites.

### 3.3 Clinical manifestations and prognostic indicators of 57 patients

The detailed characteristics of the primary tumor, including age, pathology, localization, staging, and molecular subtypes, as well as the specifics of parotid lesions, their molecular subtypes, symptoms exhibited, the presence of concomitant metastases, the interval to progression from the primary breast cancer sites to the initial diagnosis of parotid metastases, the treatment modalities employed for the metastases, and the ultimate outcomes for the cohort of 57 patients are comprehensively outlined in **Table 1**.

**Table 1:** Table summarizing the 57 cases in the literature and the patients in present study.

Case	References	Year	Age	Primary lesions						Parotid lesions				Symptoms	uDF	Concomitant metastasis	Therapy	The Outcome
				Location (breast)	Stage (TNM)	ER	PR	HER-2	Pathology	Location (parotide)	ER	PR	HER-2					
1	Katz et al. (4)	1975	49	Left	LN+	NA	NA	NA	adenocarcinoma	Right	NA	NA	NA	Mass	4 Y	Lung, bone	SP, CT	Widespread metastases, 3 M
2	Yarington et al. (50)	1981	NA	NA	NA	NA	NA	NA	adenocarcinoma	NA	NA	NA	NA	NA	NA	NA	NA	Alive, several Y
3	Wiesel, et al. (11)	1982	62	Left	Stage IV	NA	NA	NA	ILC	Left	NA	NA	NA	Mass	2 Y	Bone	RT, HT	Alive, 18 M
4			64	Left	T2N0M0	NA	NA	NA	IDC	Left	NA	NA	NA	Mass	13 Y	Bone	SP, RT	Alive, 17 M
5			74	Left	T2N2M0	NA	NA	NA	NA	Left	NA	NA	NA	Mass + FNP	8 Y	Solitary	RT, HT	Alive, 6 Y
6	Seifert, et al. (9)	1986	NA	NA	NA	NA	NA	NA	NA	NA	NA	NA	NA	NA	NA	NA	NA	NA
7			NA	NA	NA	NA	NA	NA	NA	NA	NA	NA	NA	NA	NA	NA	NA	NA
8	Bissett, et al. (19)	1989	41	Right	T2N0M0	NA	NA	NA	IDC	Left	NA	NA	NA	Mass + FNP	10 Y	Solitary	TP, RT	Alive, 2 Y
9			65	Right	T2N1M0	NA	NA	NA	NA	Left	NA	NA	NA	Mass	10 Y	Skin	RT, CT	Died, 21 M
10	Calvo, et al. (51)	1995	57	Right	NA	NA	NA	NA	IDC	Left	NA	NA	NA	Pain	NA	NA	TP	NA
11	Bochnia, et al. (20)	1997	42	Left	NA	NA	NA	NA	NA	Bilateral	NA	NA	NA	Mass + FNP	Simultaneity	Lung, mediastinum	CT, RT	Died, 2 M
12			66	Right	I	-	NA	NA	NA	Left	NA	NA	NA	Mass	4 Y	Solitary	SP, RT, HT	Died, 3 M
13	Kollias, et al. (28)	1997	52	Right	II	-	NA	NA	NA	Right	NA	NA	NA	Mass	14 Y	Solitary	SP, RT, HT	Died, 15 M
14			57	Left	II	+	NA	NA	NA	Left	NA	NA	NA	Mass	9 Y	Solitary	SP, RT, HT	Alive, 15 M
15	Joyce,et al. (7)	2000	71 (Male)	Right	T2N0M0	NA	NA	NA	IDC	Right	NA	NA	NA	Mass+FNP	15 M	NA	TP	NA
16	Lussier et al. (52)	2001	73	NA	Stage I	NA	NA	NA	IDC	NA	NA	NA	NA	Mass	2 Y	NA	NA	NA
17			67	NA	Stage I	NA	NA	NA	IDC	NA	NA	NA	NA	Only LN	2 Y	NA	NA	NA
18			56	NA	Stage I	NA	NA	NA	IDC	NA	NA	NA	NA	Mass	2 Y	NA	NA	NA
19			33	NA	Stage I	NA	NA	NA	IDC	NA	NA	NA	NA	Only LN	15 Y	NA	NA	NA
20	Szymansky, et al. (53)	2002	66	Right	NA	NA	NA	NA	IDC	Right	NA	NA	NA	FNP	NA	NA	TP	NA
21			58	Right	NA	NA	NA	NA	IDC	Right	NA	NA	NA	Mass	NA	NA	TP, RT, CT	NA
22	Zhang, et al. (12)	2003	40	Left	NA	NA	NA	NA	phyllode tumor	Right	NA	NA	NA	Mass	1.5 Y	Lung	-	Alive, 10 M
23	Nuyens et al. (13)	2006	NA	NA	pT4pN1bM0	NA	NA	NA	IDC	NA	NA	NA	NA	Mass	26 M	NA	TP, RT	Died, 18 M
24			NA	NA	pT2pN1M0	NA	NA	NA	IDC	NA	NA	NA	NA	Mass	85 M	NA	TP, RT	Died, 12 M
25	Perez, et al. (29)	2007	61	NA	T1N0M0	-	-	-	IDC	Left	+	-	NA	Mass	5 Y	Solitary	TP, RT, CT	Alive, 4 Y
26	Laforga,et al. (30)	2009	52	Left	T1N0M0	-	-	-	IDC	Left	+	-	2+	Mass	6 Y	Solitary	TP, RT, CT	Alive, 2.5 Y
27	Dangore,et al. (8)	2009	42	Right	T3N0M1	NA	NA	NA	IDC	Left	NA	NA	NA	Mass	Simultaneity	Solitary	TP, LND, RT, CT	Lost
28	Ramesh, et al. (14)	2010	63	Right	M1	+	+	-	adenocarcinoma	Right	+	+	-	Mass	Simultaneity	Solitary	HT	Alive, 3 M
29	Ando, et al. (15)	2011	69	Left	T1N3M1	-	-	3+	ILC	Left	NA	NA	3+	Mass	11 M	NA	TP, CT	Alive, 6 M
30	Cihan, et al. (38)	2011	70	Left	T2N3M0	+	+	-	IDC	Left	+	NA	-	Mass	1.5 Y	Thyroid, bones, liver	SP, RT, CT	Lost
31	Sellinger, et al. (21)	2011	74	Left	T2N1M0	+	+	-	ILC	Right	+	+	-	Mass+FNP	3 Y	Bones	TP	NA
32	Alath,et al. (3)	2014	43	Right	T2N2M1	+	+	+	IDC	Left	-	-	+	Mass	2 Y	Liver, bones	TP	Died, 7 M
33	Duncan,et al. (22)	2015	76	Right	T1N0M0	NA	NA	NA	IDC	Bilateral	+	NA	-	Mass+FNP	25 Y	Solitary	HT	NA
34	Akcan, et al. (37)	2015	61	Right	Advanced	NA	NA	NA	IDC	Right	NA	NA	NA	Mass	Simultaneity	Surrenal gland	CT+TP+surrenal gland excision	Alive, 12 M
35	Burgess, et al. (23)	2015	60	Right	NA	NA	NA	NA	IDC	Right	+	+	NA	Mass+FNP	13 Y	Bones, LN	NA	NA
36	Kmeid, et al. (16)	2016	65	Right	NA	NA	NA	NA	IDC	Right	+	NA	NA	Mass	6 Y	Solitary	TP, RT	NA
37	EI Mrabet, et al. (24)	2017	43	Right	T4dN1M0	+	+	-	inflammatory carcinoma	Left	-	-	-	Mass + FNP	2 Y	Solitary	CT	NA
38	Rawet, et al. (31)	2017	71	Left	NA	+	NA	NA	IDC	Left	+	+	-	Mass	26 Y	Solitary	HT	Alive, 5 M
39	Bohli, et al. (6)	2018	48	Right	T2N0M0	+	NA	NA	IDC	Right	NA	NA	NA	Mass	11 Y	Bone	RT, CT	Alive, 12 M
40	Cao, et al. (17)	2018	36	Left	T1N1M0	+	+	+	IDC	Left	NA	NA	NA	Mass	2.5 Y	Bones, LN, right breast	CT, HT	Alive, 6 M
41	Agrawal, et al. (32)	2018	60	Right	T2N0M0	+	+	-	IDC	Right	+	+	-	Only LN	9 M	Solitary	SP, HT	Alive, 16 M
42	Emanuelli et al. (54)	2018	55	NA	NA	NA	NA	NA	adenocarcinoma	NA	NA	NA	NA	NA	16 M	NA	TP	Died.
43	Assarian, et al. (25)	2019	54	Right	IIIIA	+	+	-	IDC	Right	+	+	+	Mass + FNP	11 M	Bone	RT, CT, TT	NA
44	Jakharia-Shah et al. (33)	2019	59	Right	T2N0M0	+	NA	+	IDC	Left	+	-	3+	Mass	8 Y	Solitary	TP, PND	Alive, 1 M
45	Razem, et al. (5)	2020	41	Right	LN+	+	-	NA	IDC	Left	NA	NA	NA	Mass	4 Y	Solitary	TP, RT, CT	Alive, 1 Y
46	Andinata, et al. (36)	2020	39	NA	T2N0M0	+	-	-	IDC	Left	NA	NA	NA	Mass	4 Y	Bone	TP, RT, CT	Progress
47	Dhia, et al. (35)	2020	50	Left	T4bN1M0	+	+	-	IDC	Bilateral	NA	NA	NA	Mass	9 Y	Lung, skin,bone, brain	CT, RT	Died, 6 M
48-9	Mayer, et al. (55)	2021 (2 cases)	75.5 (mean age)	NA	NA	NA	NA	NA	NA	NA	NA	NA	NA	NA	NA	NA	NA	NA
50	Jung HK et al. (34)	2021	59	Left	pT4N3	-	-	+	IDC	Left	-	-	+	Mass	6 Y	Contralateral axilla LN, liver, skin, bone, brain	TP, RT, CT, TT	Died, 2 M
51	Thomas et al. (18)	2021	45	Right	NA	NA	NA	NA	IDC	Right	NA	NA	NA	Mass	Simultaneity	Solitary	SP	NA
52			56	Left	NA	NA	NA	NA	IDC	Left	NA	NA	NA	Mass	Simultaneity	Solitary	SP	NA
53	King, et al. (26)	2023	35	NA	NA	NA	NA	NA	NA	Bilateral	NA	NA	NA	Enlargement + FNP	NA	Skin	CT	Progress, 6 M
54	Zhang, et al. (10)	2023	NA	NA	NA	NA	NA	NA	NA	NA	NA	NA	NA	NA	NA	NA	NA	NA
55	Peron et al. (27)	2023	69	Right	T2N3M1	-	-	3+	NA	Left	-	-	+	Mass + local pruritus	Simultaneity	Skin, LN	CT, RT, TT, MSD	Complete response, 2 Y
56	Present study	2024	58	Left	T2N1M0	+	+	-	IDC	Right	+	+	-	Mass	5 Y	Bones	TP, CT, RT, HT	Progress, 2 Y
57			72	Right	NA	+	NA	NA	IDC	Right	+	-	-	Mass	32 Y	Bones	HT	Alive, 1 Y

NA, not applicable; ILC, invasive lobular carcinoma; IDC, invasive ductal carcinoma; LN, lymph node; FNP, facial nerve palsy; RT, radiotherapy; CT, chemotherapy; HT, hormone therapy; TT, targeted therapy; SP, superficial parotidectomy; TP, total parotidectomy; PND, posterolateral neck dissection; MSD, mastectomy and sentinel LN dissection; uDF, interval to progression from primary breast sites to initial diagnosis of parotid metastases; M, months; Y, years.

Between 1975 and 2023, there have been 57 worldwide reported instances of parotid metastasis originating from breast cancer, with one notable case involving a parotid metastasis of malignant phyllodes tumor of the breast. Notably, the majority of these patients have been female, with the exception of a single male case (7). The age range of these patients has spanned from 33 to 76 years, with an average age of 57 years.

Of the 34 patients who were reported with definite presentation, the primary clinical presentation was a painless parotid gland mass. Additionally, 11 patients exhibited symptoms of facial nerve paralysis, while 1 patient complained of facial skin pruritus and another patient experienced parotid pain. Furthermore, 3 patients were diagnosed with intraparotid lymph node metastasis. Lastly, the clinical manifestations of 7 patients remained unclear.

Out of the 57 patients presenting with parotid metastasis of breast cancer, 40.4% (or 23 patients) exhibited involvement in the left parotid gland, while 29.8% (or 17 patients) demonstrated involvement in the right parotid gland. Additionally, 7.0% (or 4 patients) exhibited bilateral parotid involvement, and the involvement status for 13 patients remains unknown.

In 24 patients (42.1%), breast cancer had metastasized to the ipsilateral parotid gland, while in 4 patients (7.0%) it had metastasized to bilateral parotid glands. Additionally, 14 patients (24.6%) had metastases in the contralateral parotid gland. Of these contralateral cases, 4 patients with left breast cancer exhibited metastases in the right parotid gland, whereas 10 patients with right breast cancer displayed metastases in the left parotid gland. However, for 15 patients, information regarding the side of parotid metastasis was unavailable.

The predominant pathological subtype of primary breast cancer was invasive ductal carcinoma (IDC) in 35 patients (61.4%), followed by invasive lobular carcinoma (ILC) in 3 patients, adenocarcinoma in 4 patients, inflammatory breast cancer in 1 patient, malignant phyllode tumor in 1 patient, and unknown in 13 patients. Molecular subtyping of the primary tumor was available for only 19 patients, and in 5 patients, the metastatic lesion in the parotid gland exhibited differences from the primary breast cancer.

Immunohistochemical results were available for a total of 24 patients. In primary breast cancer, the ER status was positive among 17 patients, accounting for 70.8% of the total, while it was negative in 7 patients, representing 29.2% of the cases; PR was positive in 10 patients (58.8%) and negative in 7 patients (41.2%). HER-2 was positive in 6 patients (35.3%) and negative in 11 patients (64.7%). In the parotid metastasis of breast cancer, ER status was positive in 14 patients (82.4%) and negative in 3 patients (17.6%); PR was positive and negative in 7 patients, respectively. HER-2 was positive in 7 patients (43.8%) and negative in 9 patients (56.2%). There were 4 patients with ER/PR-, only 1 case was unifocal metastasis of parotid, and the other 3 cases were presented with concomitant metastasis in other organs. Among the patients, four individuals initially exhibited ER+ status in the primary breast cancer, yet subsequently developed ER-negative status in the metastatic parotid gland. Two patients exhibited a transition in PR status from positive to negative, while only a single patient displayed a shift in HER-2 status, transforming from negative to positive.

The metastatic status of 18 patients was unavailable. Notably, 18 patients (46.2%) exhibited merely metastasis in the parotid gland, whereas 21 patients (53.8%) presented with concurrent metastases in other organs. Among these patients with multiple metastases, 15 individuals demonstrated bone metastases. Additionally, the other metastatic sites, in descending order of frequency, encompassed the skin, lung, cervical lymph nodes, liver, mediastinum, brain, thyroid, and adrenal gland.

Seven cases exhibited concurrent occurrences of primary breast cancer and parotid metastases, while fifty cases displayed metachronous manifestations. The time elapsed between the initial diagnosis of primary breast cancer and the subsequent detection of parotid metastases was designated as uDF. Notably, the uDF was unavailable in ten patients, whereas the median uDF for the remaining patients stood at four years, ranging from a minimum of nine months to a maximum of thirty-two years.

Of the 46 patients for whom treatment strategies were known, 21 cases underwent total parotidectomy (TP), 9 cases underwent superficial parotidectomy (SP), 21 cases underwent chemotherapy, and 25 patients received radiotherapy. Of these patients who underwent surgery, 8 cases had parotid gland metastases solitary, 4 cases were concomitant with bone metastases, and 5 cases presented concomitant metastases in multiple organs. Therefore, surgeons have made patient selection based on the patients’ disease state prior to the surgery. Ultimately, of the patients who underwent surgery, 10 were alive, 7 patients succumbed to the disease and 2 patients exhibited disease progression. Thus, surgery doesn’t seem to improve the survival.

Based on the survival data outlined in the available literature, it was discovered that the survival status was unknown for 21 patients, while 2 patients were lost to follow-up. Twenty patients (accounting for 58.8% of the total) were confirmed to be alive, whereas 14 patients (41.2%) had either succumbed to their illness or experienced disease progression. Furthermore, there were significant disparities in the characteristics of these two patient cohorts.

Of the patients who were still alive, the median uDF was 5.5 years, ranging from 0 to 32 years. Similarly, the median survival time among these patients was 15 months, with a range extending from 1 month to 6 years. Fourteen patients, representing 70% of the total, exhibited metastasis to the ipsilateral parotid gland. Five patients, accounting for 25%, showed metastasis to the contralateral parotid gland, while the metastatic status of one patient remained unknown. Ten patients, or 50% of the total, exclusively harbored parotid gland metastasis. Eight patients, constituting 40%, presented with metastasis to other organs, including five patients with bone metastasis alone. The metastatic status of two patients, however, were unknown.

Among those who died or progressed, 10 patients succumbed to the illness, while another 4 developed progression or widespread metastases. The median uDF was 5.0 years, ranging from 0 to 14 years, with a median time to either death or disease progression of 6 months(2 months-2 years). Among the patients, contralateral or bilateral parotid metastases were detected in 8 cases (accounting for 57.1% of the total), ipsilateral parotid metastases in 2 cases, and unknown in 4 cases. Only 2 patients (14.3%) presented with parotid metastases exclusively, whereas 9 patients (64.3%) exhibited metastases in other organs as well, and the status of 3 patients was undetermined.

**Figure 5:**
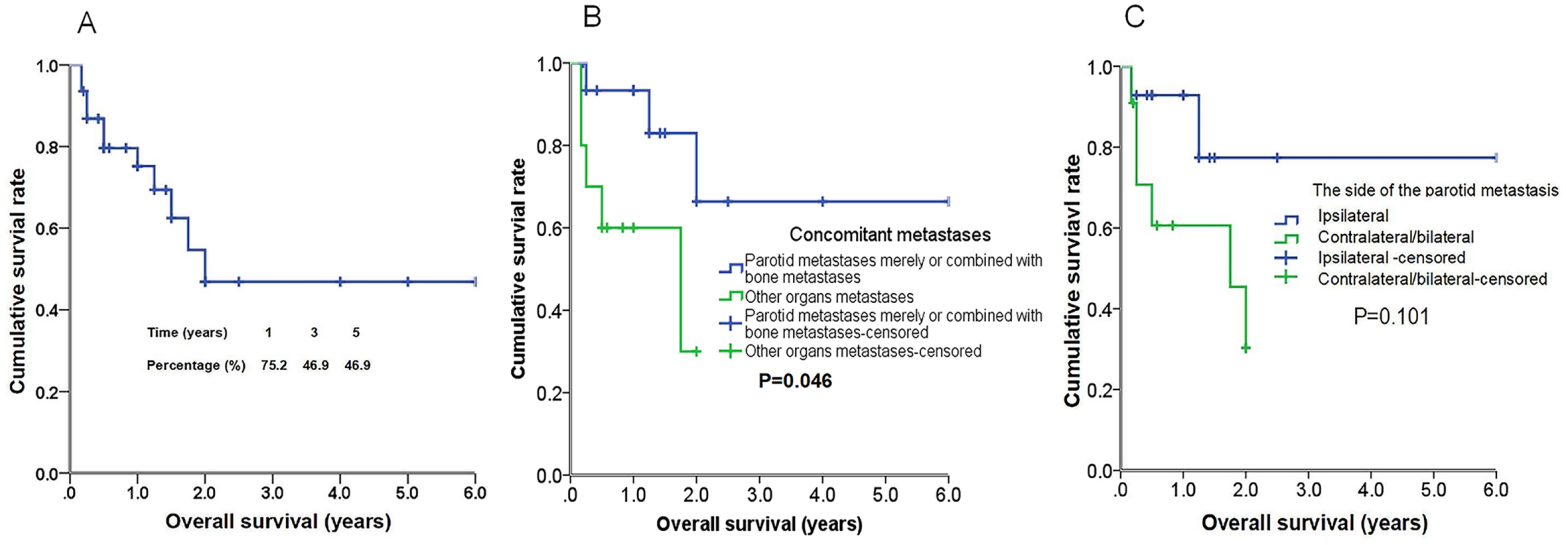
Kaplan-Meier curves of overall survival (OS) of 57 patients with parotid metastasis of breast cancer **(A)**. Kaplan–Meier curves of OS in patients with parotid metastases merely or combined with bone metastases versus patients with other organs metastases **(B)** for OS, and the differences between groups were significant (*P*<0.05, log-rank test). Kaplan–Meier curves of OS in patients with ipsilateral parotid metastases versus contralateral or bilateral parotid metastases **(C)** for OS, and the differences between groups were not significant (*P* > 0.05, log-rank test).

A subsequent analysis was conducted to identify the prognostic factors associated with parotid metastasis in breast cancer patients. The cumulative survival curve, presented in **Figure 3**, provides a comprehensive overview of the survival outcomes for 57 patients with this condition. Notably, patients who exhibited parotid metastases alone or combined with bone metastases exhibited significantly longer overall survival compared to those with concurrent metastases in other organs (1.23 ± 0.26 years vs 4.46 ± 0.77 years, P=0.046). The Kaplan-Meier curve, depicted in **Figure 5A**, further illustrates these survival patterns. Patients with metastases to the contralateral or bilateral parotid gland exhibited a mean OS of 1.29 ± 0.28 years, whereas those without such metastases had a significantly longer mean OS of 4.85 ± 0.76 years (P=0.101) (**Figure 5B**). Although statistical significance was not reached, a notable difference in OS between the two groups was observable from the Kaplan–Meier curve (**Figure 5C**). Furthermore, neither the presence of different uDFs nor the type of treatment received (surgical or palliative) had a significant impact on survival rates. The detailed results are summarized in **Table 2**.

**Table 2:** Univariate analyses of predictors of overall survival.

Characteristic	Univariate Kaplan-Meier analyses		
	$\bar{x}$ ± SD (years)	*x^2^*	*P*
The side of the parotid metastasis of breast cancer		2.69	0.101
Contralateral/bilateral	1.29 ± 0.28		
Ipsilateral	4.85 ± 0.76		
uDF		0.20	0.659
≤ 6 years	2.49 ± 0.47		
> 6 years	3.69 ± 1.02		
Concomitant metastases		3.98	0.046
Parotid metastases merely or combined with bone metastases	4.46 ± 0.77		
Other organs metastases	1.23 ± 0.26		
Treatment		0.25	0.618
TP/SP	2.20 ± 0.46		
Palliative	3.58 ± 1.02		

*P <0.05, SP, Superficial Parotidectomy; TP, Total Parotidectomy; uDF, Interval to progression from primary breast sites to initial diagnosis of parotid metastases.

## 4 Discussion

The most common sites for breast cancer metastases include the lungs, liver, bone, adrenals and brain with rare cases disseminated to head and neck (8). Although rarely encountered, metastatic disease to the parotid glands was occasionally reported in the medical literature as isolated case reports or small series. Siefert et al. (9) conducted a retrospective analysis on 10,944 patients harboring parotid gland tumors, revealing that only 75 of these cases were metastatic from primary cancers in other organs. Notably, just two of these metastatic instances originated from breast cancer. In a more recent study, Zhang et al. (10) analyzed data from 6274 consecutive patients with stage IV breast cancer who had developed unusual metastases. Surprisingly, only a single case involved the parotid gland. Given the rarity of this particular metastasis, there remains a significant knowledge gap in understanding its etiology and pathophysiology.

The clinical presentation of parotid metastases were mostly painless masses (4, 8, 11–18), solitary or multifocal, some patients associated with facial nerve paralysis (7, 11, 19–25), and a few patients had special manifestations. King et al. (26) documented an occurrence of unique patient presenting with synchronous bilateral facial nerve paralysis secondary to metastatic breast carcinoma to the bilateral parotid glands, while the CT scan of neck demonstrated heterogenous enhancement and enlargement of the bilateral parotid glands, rather than the presence of distinct masses. Additionally, a patient was reported with have complained of parotid mass accompanied by local pruritus (27). The diversity of symptoms observed in these cases has been hypothesized to be associated with the malignant nature of the underlying condition (16).

Breast cancer can present with parotid gland metastasis merely (5, 8, 11, 14, 16, 18, 24, 28–33), or it can presents with synchronous multiple metastases of other organs (12, 27, 34–37), including bone, lung, mediastinum, brain, lymph node, adrenal gland, etc. Bones are particularly susceptible to metastatic involvement. In addition, metastasis in other rare sites can also be noted. Cao et al. (17) and Jung HK et al. (34) described instances where breast cancer metastasized to the parotid gland, accompanied by concurrent metastasis to the contralateral breast and contralateral axillary lymph nodes, respectively. Cihan et al. (38) reported a patient who exhibited synchronous metastasis to both the parotid and thyroid glands originating from breast cancer.

Breast cancer metastases not only to the ipsilateral parotid gland, but also to the contralateral and bilateral parotid glands. Of the 44 patients with known sides of parotid gland metastases, 40.9% (18 out of 44) exhibited either contralateral or bilateral metastases. Katz et al. (4) firstly reported a metastatic adenocarcinomarcinoma from the left breast to the right parotid gland in 1975. Since then, a mere 13 additional instances have been reported where the breast cancer has involved the contralateral parotid gland. Notably, only four cases have been documented with bilateral parotid metastases (20, 22, 26, 35). Nevertheless, due to the rarity of this metastatic variation, the precise metastatic pathway remains elusive.

No matter the patients with breast cancer metastasis to the ipsilateral parotid, or to the contralateral or bilateral parotid, non-involvement of axillary nodes was identified, indicating that hematogenous dissemination appears to be the predominant mode of spread, rather than direct lymphatic metastasis. This assertion is corroborated by prior research, which has reported similar frequencies of metastases to the ipsilateral and contralateral parotid glands from breast cancer (15, 16, 33). Additionally, the occurrence of metastases in the contralateral and bilateral parotid glands may serve as predictors of a poor prognosis.

Invasive ductal carcinoma is the most commonly reported pathological subtype of primary breast cancer in the extant literature, as evidenced by the majority of studies (5, 8, 17, 18, 25, 33–36, 39), which means that the most likely culprit of parotid mets is actually the most common form of breast cancer. It is of utmost importance for head/neck surgeons to maintain a high index of suspicion for parotid gland metastasis stemming from breast cancer in patients presenting with a parotid gland tumor and a prior history of invasive ductal carcinoma of the breast. Additionally, metastases arising from other types of breast cancer, including invasive lobular carcinoma, inflammatory carcinoma, and even malignant phyllodes tumor, have also been described (12, 15, 21, 24). While primary malignancies of the parotid glands can exhibit sex hormone receptors, the majority tend to express androgen receptors, with only a minority expressing oestrogen receptors (40). Similarities between salivary ductal carcinoma of the parotid gland and metastatic breast carcinoma, make it difficult to distinguish metastasis from primary carcinoma. During the process of breast cancer metastasis to the parotid gland, the immunohistochemistry profiles of the patient changed, resulting in different molecular subtype from primary site (3, 24, 25, 30). Previous studies have reported divergent expression of hormonal receptors between primary tumors and metastatic sites (41). Fine needle aspiration cytology remains the gold standard for accurately distinguishing between primary and secondary tumors, with an accuracy rate of 85% (16, 33). This technique plays a crucial role in the diagnosis and management of patients with breast cancer metastasis to the parotid gland.

A differential diagnosis is salivary duct carcinoma (SDC), which is with morphological resemblance to IDC of the breast. The main discrepancy between IDC and SDC in the immunohistochemical profiles was a distinctly different absence in ER-α, PR and HER-2 (42). Positive reactivity to ER-α, PR or both and negative HER-2 favors a diagnosis of IDC while ER-α, PR negative, HER-2 positive tumors are more likely SDC. However, for patients present with unifocal lesion in parotid and ER/PR negative, accurate identification of the primary tumor is particularly difficult in morphology. SDC has a male predilection and mostly occurs in elderly patients in clinical, which is a distinctly different with IDC. The differential diagnosis should be made based on the patient’s medical history, sex, age, and immunohistochemistry.

The interval between the diagnosis of primary breast cancer and the development of distant metastases is quite variable. In fact, distant metastases can occur up to several decades from the original diagnosis (43, 44). A latest study (10), the researchers discovered that the median duration between the initial diagnosis of primary breast cancer and the detection of unusual metastatic occurrences was approximately 121.3 months. The median uDF for the entire cohort of 57 patients was 4 years, with a remarkable outlier of one patient who exhibited a protracted uDF period extending as long as 32 years. The preponderance of metastatic cells often remains in a quiescent state for extended durations, referred to as metastatic dormancy. However, these cells can subsequently become activated and proliferate, ultimately leading to the manifestation of pronounced metastases (45–47). Given that 5-year freedom from recurrence does not guarantee long-term disease-free survival, the sustained surveillance of these patients is of paramount importance.

In cases where the metastasis is confined to the parotid gland, the management remains palliative. However, an appropriate parotidectomy with negative margins and preservation of the facial nerve should be carefully considered to alleviate clinical symptoms and contain local disease progression (15, 46).

Consequent to their rarity, no consensus was achieved on treatment protocol in the metastatic cancer scenario involving the parotid gland. For instances of single parotid gland metastasis originating from breast cancer, previous studies have primarily suggested surgeries such as superficial or total parotidectomy, with preservation of facial nerves to achieve negative margin in previous studies (6, 15, 16). In the case involving multiple-site metastases, the recommended approach often involves systemic therapy, encompassing chemotherapy, hormonal therapy, and possibly a combination with radiation therapy (17). Despite the proposed treatments, parotid surgery dose not seem to improve survival, patients with metastatic involvement of the parotid gland have poor prognosis, with the 5-year survival rate of only 10% (16, 17). Of the 30 patients undergoing surgery for parotid metastatic breast cancer, 12 patients were found to have metastasis solely to the parotid gland or in combination with bone metastases. Despite careful patient selection by medical professionals prior to surgery, surgical intervention did not seem to significantly enhance survival rates. Although the management of a parotid metastasis is palliative, in cases where the metastasis is merely to the parotid gland, it is advisable to consider an appropriate parotidectomy that ensures negative margins and preservation of the facial nerve, to relieve clinical symptoms and control local progression (16, 48). It is crucial to tailor the management approach based on the individual patient’s condition and the progression of their disease.

Survival rates can be influenced by several factors, including the clinical presentation of the disease, the duration between the initial diagnosis of breast cancer and the emergence of distant metastases, as well as the specific location of recurrence. Previous research underscores the notion that patients with metachronous solitary parotid metastases and a prolonged disease-free survival period are regarded as having favorable prognostic indicators (49). Additionally, those individuals who exhibit a longer uDF and present with non-visceral metastatic deposits are generally predisposed to more favorable outcomes. Patients who present with parotid-only metastases or those combined with bone-only metastases tend to enjoy a significantly more favorable prognosis. While the available survival data in the literature remains limited, our findings reveal that these subsets of patients fare much better in terms of survival compared to those who suffer from metastases in other organs. This observation is further corroborated by prior studies, which indicate that individuals who solely develop rare metastasis or bone-only metastasis enjoy a significantly superior prognosis compared to those who also have common visceral metastasis (10).

Furthermore, among patients who survived, the uDF was extended compared to those who either succumbed to the disease or experienced disease progression(5.5 years vs 5.0 years), and 70% (14 out of 20) displayed metastases to the ipsilateral parotid gland. Of the patients who died or experienced disease progression, a notable 57.1% (8 out of 14) exhibited metastasis in the contralateral or bilateral parotid glands, while only two individuals exhibited metastasis in the ipsilateral parotid gland. Despite the statistical analysis failing to uncover a statistically significant difference in survival rates between patients with ipsilateral and contralateral or bilateral parotid metastases, the Kaplan-Meir curve revealed a marked divergence between the two groups. The survival rates of patients who were alive were grossly underestimated due to inconsistencies in the censoring dates of survival data reported in the literature. However, the survival outcomes of patients who succumbed to the disease or experienced disease progression were unambiguous. Consequently, by obtaining comprehensive survival data for these alive patients, we anticipate that additional factors influencing the prognosis of patients with parotid metastasis from breast cancer may be uncovered.

As a retrospective study, limitations can’t be neglected. Firstly, the data were derived from literature review, which led to an inevitable heterogeneity; Secondly, the grouping is not detailed enough due to the sample size limitation; thirdly, the censoring dates of survival were different in the literature, resulting in inaccurate survival time. Nevertheless, the clinical characteristics and prognosis predictors of parotid gland metastases from breast cancer in present study, remains valid.

## 5 Conclusion

In conclusion, parotid gland metastases from breast origin are extremely rare. Despite its low incidence, its unique presentation, biological behavior, and prognostic factors can be found from the literature review. Parotid gland metastases can occur synchronously or metachronously, involve ipsilateral, contralateral or bilateral parotid glands, or coexist with concomitant metastasis in multiple organs. Notably, patients with parotid-only metastases or those with combined bone-only metastases, as well as those with ipsilateral parotid metastases, tend to have more favorable prognostic outcomes. Consequently, it is imperative to exercise greater vigilance in monitoring patients who exhibit metastasis in other organs, as well as those with contralateral or bilateral parotid metastasis.

## Data Availability

The original contributions presented in the study are included in the article/supplementary material. Further inquiries can be directed to the corresponding authors.
